# Neuron‐glia crosstalk shapes brain morphogenesis

**DOI:** 10.1002/ctm2.1004

**Published:** 2022-07-31

**Authors:** Laurent Nguyen

**Affiliations:** ^1^ Laboratory of Molecular Regulation of Neurogenesis, GIGA‐Stem Cells and GIGA‐Neurosciences, Interdisciplinary Cluster for Applied Genoproteomics (GIGA‐R) University of Liège, CHU Sart Tilman Liège Belgium

1

The cerebral cortex has a complex cytoarchitectonic characterized by cellular layers tangentially organized into distinct areas that compute higher cognitive functions. Its morphogenesis relies on the rearrangement of a primordial structure that progresses through successive steps, ranging from neurogenesis to synaptogenesis. With form comes function and disruption of some developmental steps can lead to cortical malformations associated with a wide spectrum of clinical presentations. The cellular and molecular mechanisms underlying cerebral cortex morphogenesis are intricate and the exquisite cellular layout of the cortical wall results from the orchestrated migration of neural precursors born in distinct germinative forebrain regions.

Cell migration not only brings precursors to their final position in the cortex but also promotes transient interactions with other cells, thereby conferring additional roles to those played once integrated into the cortical network.^1^ This is exemplified by recent works showing that cell migration conveys key instructive cues to neighbouring cells in the developing cortex.^2^, ^3^, ^4^, ^5^ Cortical neurons, which comprise interneurons (cINs) born in the subpallium and projection neurons (PNs) generated in the pallium, undergo migration to settle in ad hoc cortical layers where they functionally integrate circuits. The cINs migrate along tangential paths to reach the cortex, a process during which they engage in cellular crosstalk and read extracellular cues to find their way. These neurons are attracted to the cortex by a gradient of the chemokine Cxcl12 released by cortical progenitors and cINs signal back to those progenitors to regulate their amplification, thereby the output of the upper layer PNs.^5^, ^6^ Moreover, the interaction between cINs and PNs regulates cINs survival and provides them with key spatial information to settle into the cortex.^7^, ^8^ Recent work showed that reduction in microglia number results in impaired positioning of cINs in the developing cortex,^2^ supporting a possible role for glia in neuron guidance.

Along this line, our laboratory discovered that some oligodendrocyte precursor cells (OPCs) steer the migration of cINs in the forebrain via direct contact.^4^ Cerebral OPCs are generated in three competitive waves: two initial ones, born during embryogenesis in the ganglionic eminences and the preoptic area, and a third one that arises from cortical progenitors around birth.^9^ Third‐wave OPCs generate most oligodendrocytes that myelinate later PN axons while some 1st wave ventrally‐derived OPCs (vOPCs) form GABAergic synapses with lineage‐related cINs^10^ and later contribute to cIN myelination.^11^ Interestingly, cINs and 1^st^ wave vOPCs are born in overlapping germinative compartments and migrate together in the forebrain to reach the developing cortical wall. We thus assessed whether these cell populations may crosstalk during embryogenesis. While cINs navigate within the cortex in organized streams, 1st wave vOPCs undergo random walking and later disperse via dynamic gliding along the blood vessels (BVs) that are growing in the forebrain.^4^, ^12^ The conditional elimination of vOPCs results in a less cortical invasion by cINs. Both vOPCs and cINs are attracted by Cxcl12 locally released by BVs, but 1st wave vOPCs are the ones covering BVs because they prevent cINs to interact with BVs via unidirectional contact repulsion (UCoRe). Therefore, loss of 1st wave vOPCs leads to the accumulation of cINs in the vicinity of BVs, where they tend to aggregate. UCoRe is a repulsive signalling mediated by the interaction between PlexinA3 (expressed by cINs) and Sema6a/b (by 1st wave vOPCs), which causes cINs to retract their leading process, change polarity and migrate away from the 1st wave vOPCs.^4^ This cell behaviour can be compared to a tennis ball (cIN) bouncing back on the opponent's racket (1st wave vOPC) during a volley shot.

Here, we unravelled a transient and non‐canonical role for 1st wave vOPCs – whose number is greatly reduced after birth by apoptosis – in guiding cIN migration during embryogenesis. Class A plexins and their ligands are implicated in various aspects of cortical development and our study demonstrates how some axon guidance cues are reused in a different biological context. The idea that some migrating precursors rely on each other for reaching their individual target areas is likely to apply more generally. Collectively, these data show that the establishment of cellular crosstalk between migrating precursors is required for proper brain morphogenesis. Importantly, accumulating evidence shows that disruption of some of these crosstalks or the emergence of pathological ones contribute to the onset and progression of neurological disorders.^13^, ^14^
